# Understanding Drug Interactions in Antiplatelet Therapy for Atherosclerotic Vascular Disease: A Systematic Review

**DOI:** 10.1111/cns.70258

**Published:** 2025-02-09

**Authors:** Xiangqian Huang, Jiahao Song, Xiaoming Zhang, Mengqi Wang, Yuchuan Ding, Xunming Ji, Da Zhou, Ran Meng

**Affiliations:** ^1^ Department of Neurology Xuanwu Hospital, Capital Medical University Beijing China; ^2^ Advanced Center of Stroke Beijing Institute for Brain Disorders Beijing China; ^3^ Department of Neurosurgery Wayne State University School of Medicine Detroit Michigan USA

**Keywords:** antiplatelet drugs, atherosclerotic vascular disease, drug–drug interactions, personalized therapy

## Abstract

**Background:**

Antiplatelet drugs are a cornerstone in managing atherosclerotic vascular disease (ASVD). However, their interactions with other medications present significant challenges to treatment efficacy and safety. Patients with ASVD often require multiple treatment regimens due to complex comorbidities, which increases the risk of drug–drug interactions (DDIs). These interactions can lead to drug resistance, reduced therapeutic outcomes, or adverse effects. A thorough understanding of DDIs is crucial for optimizing patient care.

**Aims:**

This review aims to explore the clinical significance. mechanisms, and implications of DDIs in antiplatelet therapy Additionally, it seeks to identify future research directions to advance personalized treatment strategies and improve therapeutic outcomes.

**Materials and Methods:**

A systematic literature review was conducted using key databases, focusing on clinical studies, mechanistic research, and guidelines related to antiplatelet therapy and DDIs. Findings were analyzed to identify common interaction patterns, associated risks, and management strategies.

**Results:**

The review identifies common DDIs involving antiplatelet drugs, particularly with anticoagulants, nonsteroidal anti‐inflammatory drugs, and proton pump inhibitors. These interactions primarily occur through pharmacokinetic mechanisms, such as alterations in drug metabolism via cytochrome P450 enzymes, and pharmacodynamic mechanisms, including synergistic or antagonistic effects on platelet inhibition. Clinically, DDIs can increase bleeding risk, reduce antiplatelet efficacy, and contribute to adverse cardiovascular outcomes. Strategies to mitigate these risks include individualized drug selection, dose adjustments, genetic testing, and therapeutic drug monitoring.

**Discussion:**

Effective management of DDIs in antiplatelet therapy is essential to improve clinical outcomes. A patient‐specific approach, considering comorbidities, genetic predispositions, and concurrent medications, is crucial. The review categorizes DDIs based on clinical settings and underscores the need for further research on predictive biomarkers, pharmacogenomics, and advanced monitoring techniques.

**Conclusion:**

DDIs significantly impact the effectiveness and safety of antiplatelet therapy, necessitating a comprehensive understanding of their mechanisms and clinical implications. Future research should focus on developing personalized treatment approaches, integrating genetic testing, and optimizing pharmacological monitoring to minimize risks and improve therapeutic outcomes. This review provides a foundation for advancing clinical practice and enhancing the management of patients with ASVD.

## Graphical abstract

Antiplatelet drugs are essential for treating atherosclerotic vascular disease (ASVD), but drug interactions can lead to resistance and affect therapeutic efficacy. Due to the complexity of treatments in ASVD patients, understanding these interactions is critical for optimizing therapy and reducing adverse effects. This article examines the clinical implications of these interactions and highlights potential research directions for individualized antiplatelet strategies.

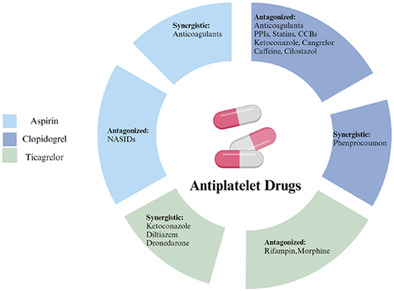

## 1 Introduction

Atherosclerotic vascular disease (ASVD) remains the leading global cause of disability and mortality, necessitating a multifaceted approach for its treatment and prevention due to its heterogeneous and multifactorial nature [1, 2, 3, 4, 5]. Dual antiplatelet therapy (DAPT), which combines aspirin with a P2Y12 receptor inhibitor such as clopidogrel or ticagrelor, is a cornerstone of early ASVD management [6].

Despite its clinical utility, resistance to antiplatelet drugs frequently occurs, driven by genetic, pharmacological, or clinical factors. This underscores the critical need for clinicians to understand the pharmacodynamics and pharmacokinetics of these therapies, with particular emphasis on drug–drug interactions (DDIs). DDIs arise when one medication alters the activity, efficacy, or toxicity of another, leading to reduced therapeutic effectiveness or unexpected adverse effects. In ASVD management, polypharmacy is common, as patients are often treated for comorbid conditions such as hypertension, hyperlipidemia, and diabetes in addition to receiving antiplatelet or anticoagulant therapies. This complex pharmacological landscape heightens the risk of DDIs, which can compromise therapeutic outcomes, exacerbate bleeding risks, impair drug efficacy, or induce organ toxicity. Drug interactions are typically categorized into three types: (1) Pharmacodynamic interactions, which involve the interplay of drugs with similar or opposing mechanisms of action. For instance, two drugs with cumulative effects may amplify therapeutic benefits or increase adverse side effects. (2) Pharmacokinetic interactions, where one drug alters the absorption, distribution, metabolism, or excretion of another, potentially leading to subtherapeutic or toxic drug levels. For example, interactions involving cytochrome P450 enzymes may result in altered plasma drug concentrations and efficacy. (3) Clinical interactions, observed in real‐world practice, where the overall impact of DDIs is reflected in patient outcomes.

This review systematically examines the current evidence on DDIs in antiplatelet therapy, with a focus on aspirin, clopidogrel, prasugrel, and ticagrelor. By summarizing recent findings, we aim to provide a comprehensive understanding of these interactions and their implications for clinical practice, paving the way for more effective and personalized therapeutic strategies in ASVD management.

## 2 Methods

### 2.1 Search Strategy

A systematic review of the literature was conducted using PubMed Central (PMC) and EMBASE databases. The search employed the keywords “antiplatelet drug,” “drug–drug interaction,” “Major Adverse Cardiovascular Events,” and “Bleeding events.” Articles published up to October 2024 were included in the analysis.

The review adhered to the Cochrane Handbook for Systematic Reviews of Interventions and followed the guidelines outlined in the Preferred Reporting Items for Systematic Review and Meta‐Analysis Protocols (PRISMA‐P).

### 2.2 Study Selection

The inclusion and exclusion criteria for study selection were as follows:

**Inclusion criteria:**

1. Studies examining DDIs in patients with ASVD or related conditions (e.g., ischemic stroke, myocardial infarction);
2. Articles addressing therapeutic drug interactions involving antiplatelet agents (e.g., aspirin, clopidogrel, prasugrel, and ticagrelor) and other cardiovascular drugs;
3. Clinical trials, cohort studies, case‐control studies, observational studies, and meta‐analyses reporting relevant outcomes.

**Exclusion criteria:**

1. Studies not specifically focused on ASVD or related conditions;
2. Articles published in languages other than English;
3. Studies without full‐text availability or access;
4. Conference abstracts, reviews, case reports, and letters.

**FIGURE 1:**
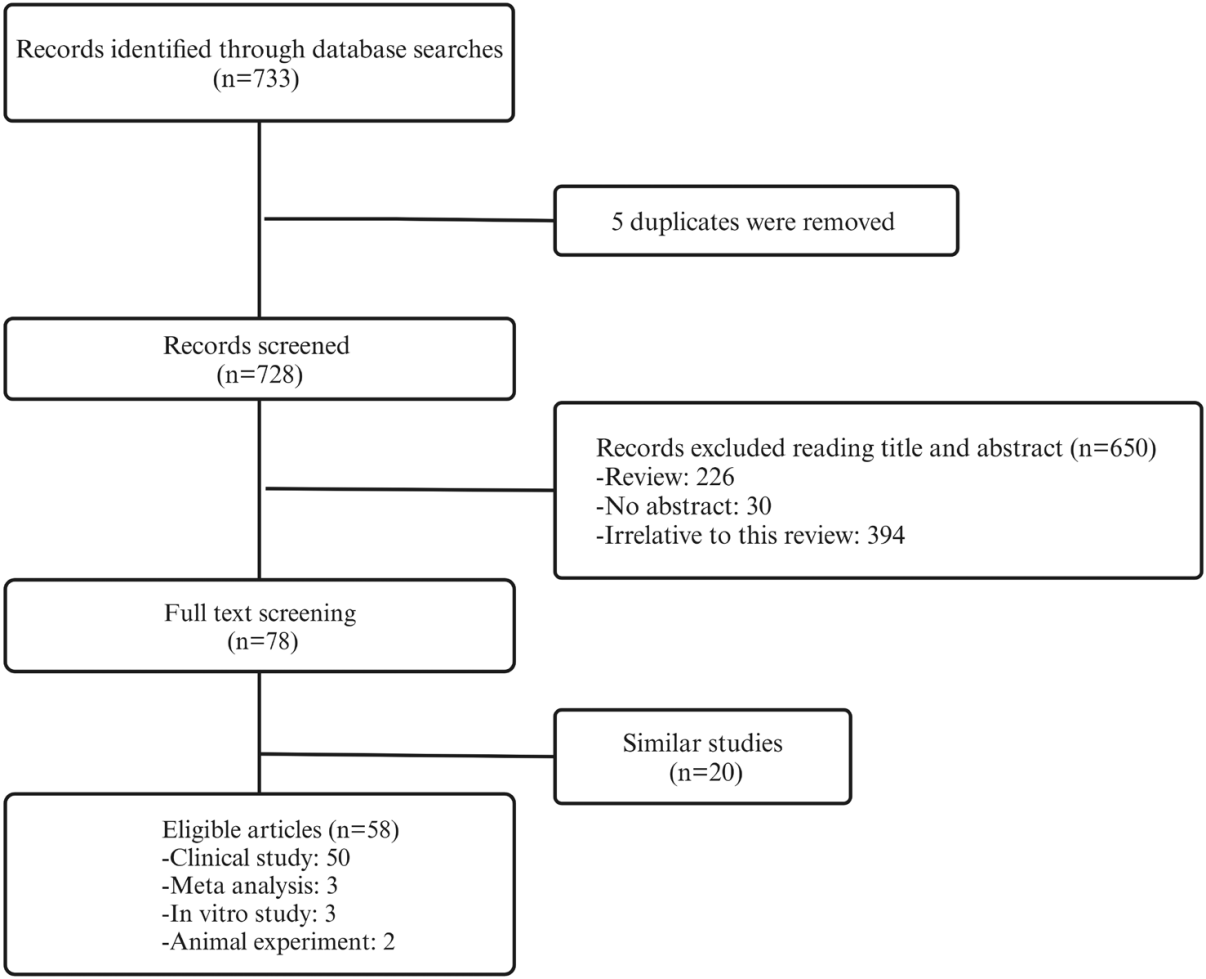
Flow diagram of the study selection process.

In cases where studies contained duplicate or overlapping data, the study with the larger sample size and more comprehensive data was selected. One reviewer independently performed the study selection, and any disagreements were resolved through discussion with a second reviewer. The detailed screening process is illustrated in Figure 1.

### 2.3 Data Extraction

Data extraction was conducted by one author (XQ‐H) and subsequently validated by another author (D‐Z). Extracted data included the name of the first author, year of publication, country of origin, research design and subject characteristics, antiplatelet drugs studied and concomitant medications, and main findings.

### 2.4 Outcomes

The primary outcomes analyzed in this review were the rate of cardiovascular and cerebrovascular events or platelet activation rates and the incidence of bleeding events.

### 2.5 Results

A total of 58 studies met the inclusion criteria, comprising 50 clinical studies, 3 meta‐analyses, 3 in vitro studies, and 2 animal experiments. The specific screening process is shown in Figure 1, and detailed characteristics of the included studies are provided in Table 1.

**TABLE 1:** Drug–drug interaction in antiplatelet drugs.

Reference	Year	Country	Study type	Subject	Antiplatelet drugs	Combined drugs	Main findings
Schaefer et al. [23]	2019	USA	Case–control	Patients receiving warfarin therapy and aspirin	Aspirin	Warfarin	Compared with warfarin monotherapy, receipt of combination warfarin and aspirin therapy was associated with increased bleeding
Yi et al. [24]	2024	USA	Retro	Patients after LVAD placement	Aspirin	Warfarin	The odds of a bleeding event occurring were higher in the warfarin and aspirin group
Schaefer et al. [29]	2021	USA	Case–control	Adults undergoing treatment with a DOAC for AF or VTE	Aspirin	DOACs	Compared with DOAC monotherapy, concurrent DOAC and aspirin use was associated with increased bleeding and hospitalizations but similar observed thrombosis rate
Awa et al. [31]	2012	Japan	Case–control	In vitro study	Aspirin	Ibuprofen	The lower OTC dose of ibuprofen (150 mg) was enough to affect the antiplatelet effect of aspirin
Galliard‐Grigioni et al. [32]	2009	Switzerland	Case–control	Volunteer	Aspirin	NSAID	A coadministration of NSAID and aspirin may interfere with platelet inhibition at the beginning of a treatment with an increase of naproxen and a decrease of diclofenac
Zhang et al. [38]	2019	China	Case–control	Rat	Aspirin	Amoxicillin	The metabolic activity of aspirin through the intestinal flora is slowed down after administration of amoxicillin
Axelsson et al. [40]	2024	Sweden	Meta‐analysis	NA	Aspirin	SSRIs	Low‐dose aspirin added to an SSRI may imply an increased risk of bleeding primarily attributable to the initiation of aspirin
AI‐Musawe et al. [41]	2020	Portugal	Case–control	Elderly people with type 2 diabetes	Aspirin	SSRIs	Aspirin combined with selective serotonin reuptake inhibitors (SSRIs) has potential drug–drug interactions
Serebruany et al. [42]	2003	USA	Case–control	Patients with ACS	Aspirin	Sertraline	Treatment with sertraline in depressed post‐ACS patients is associated with reductions in platelet/endothelial activation despite coadministration of widespread antiplatelet regimens including aspirin and clopidogrel
Serebruany et al. [43]	2005	USA	Case–control	Patients with ACS	Aspirin	Sertraline	Plasma levels of sertraline and its primary metabolite N‐desmethylsertraline could affect the release of platelet/endothelial biomarkers
Sibbing et al. [49]	2010	Germany	Case–control	Patients under dual antiplatelet treatment	Clopidogrel	Phenprocoumon	Phenprocoumon significantly attenuates the antiplatelet effects of clopidogrel
Gremmel et al. [50]	2010	Austria	Case–control	Patients after PCI	Clopidogrel	CCBs	CCBs decrease clopidogrel‐mediated platelet inhibition in patients undergoing angioplasty and stenting for cardiovascular disease
Siller‐Matula et al. [51]	2008	Austria	Case–control	Patients after PCI	Clopidogrel	CCBs	Calcium‐channel blockers reduce the antiplatelet effect of clopidogrel
Park et al. [52]	2012	Korea	Case–control	Patients with successful genotyping of CYP3A5	Clopidogrel	CCBs	Treatment with amlodipine is associated with increased clopidogrel OPR and increased risk of thrombotic events after PCI, which is dependent on the CYP3A5 genetic status
Seo et al. [53]	2014	Korea	Case–control	Patients with cerebrovascular disease	Clopidogrel	CCBs	CCBs inhibit the antiplatelet activity of clopidogrel
Park et al. [54]	2013	Korea	Case–control	Patients with drug‐eluting stent implantation	Clopidogrel	CCBs	The number of CYP3A4 (IVS10+12G>A) A‐alleles may be associated with an increased vulnerability to the effects of CCBs on clopidogrel response variation
Good et al. [57]	2012	USA	Case–control	Patient planned for PCI	Clopidogrel	CCBs	There was no evidence that CCBs decrease the efficacy of clopidogrel
Maret‐Ouda et al. [60]	2022	Sweden	Case–control	Patients who received clopidogrel after PCI	Clopidogrel	PPI	Concomitant use of PPI seems to increase the risk of major cardiovascular events
Lee et al. [61]	2023	Korea	Self‐control	Patients with stroke and myocardial infarction	Clopidogrel	PPI	A PPI coprescription > 4 weeks with clopidogrel was associated with hospitalization of recurrent stroke within 1 year of initial diagnosis
Chang et al. [62]	2024	China	Retro	Patients with ACS	Clopidogrel	Omeprazole	Patients taking both clopidogrel and omeprazole were associated with an increased risk of IS
Luo et al. [63]	2022	China	Meta‐analysis	Patients with CHD	Clopidogrel	PPI	The concomitant use of PPIs with aspirin and clopidogrel was associated with a reduced risk of GI complications but could increase the rates of MACEs
Ohbuchi et al. [64]	2012	Japan	Case–control	In vitro study	Clopidogrel	PPI Famotidine	CYP2C19 inhibition is the main cause of drug–drug interaction between clopidogrel and omeprazole. Famotidine is considered as a safe anti‐acid agent for patients taking clopidogrel
Furuta et al. [67]	2010	Japan	Case–control	Healthy volunteers	Clopidogrel	PPI	In rapid metabolizers of CYP2C19, omeprazole and rabeprazole significantly attenuated the anti‐platelet function of clopidogrel. In decreased metabolizers, there was a large variation in IPA and there was a trend but no significant decrease in IPA when placed on a concomitant PPI
Arbel et al. [68]	2013	Israel	Case–control	Patients treated with aspirin and clopidogrel	Clopidogrel	Omeprazole Pantoprazole Famotidine	Omeprazole therapy was associated with substantially more HPR than famotidine or pantoprazole
Wenaweser et al. [70]	2010	Switzerland	Case–control	Patients with dual antiplatelet therapy with aspirin and clopidogrel	Aspirin Clopidogrel	Atorvastatin Fluvastatin	Neither atorvastatin 40 mg daily nor fluvastatin 80 mg daily administered in combination with standard dual antiplatelet therapy following coronary drug‐eluting stent implantation significantly interfere with the antiaggregatory effect of ASA and clopidogrel
Serrano et al. [71]	2010	Brazil	Case–control	Patients with stable angina	Clopidogrel	Simvastatin Atorvastatin	Clopidogrel has no antiplatelet effect reduced in the presence of simvastatin or atorvastatin
Karaźniewicz‐Łada et al. [72]	2011	Poland	Case–control	Patients after coronary angiography/angioplasty	Clopidogrel	Atorvastatin Rosuvastatin	The CYP2C19*2 allele is the primary determinant of the exposition to the H4 active metabolite of clopidogrel and platelet reactivity in patients cotreated with atorvastatin or rosuvastatin
Lau et al. [73]	2003	USA	Case–control	Patients undergoing coronary artery stent implantation	Clopidogrel	Statin	Use of a statin not metabolized by CYP3A4 and point‐of‐care platelet function testing may be warranted in patients treated with clopidogrel
Wang et al. [74]	2015	China	Case–control	In vitro study	Clopidogrel	Simvastatin	The inhibitory effect of simvastatin on the hydrolysis of clopidogrel and its principal bolites may have offset the influence of simvastatin‐mediated inhibition of CYP3A4, and permitted the unaltered formation of the clopidogrel active metabolite
Farid et al. [76]	2007	USA	Case–control	Healthy subjects	Prasugrel Clopidogrel	Ketoconazole	CYP3A4 and CYP3A5 inhibition by ketoconazole affects formation of clopidogrel's but not prasugrel's active metabolite
Judge et al. [77]	2010	UK	Case–control	Healthy volunteers	Clopidogrel	Rifampicin	Potentiation of clopidogrel AM production by rifampicin leads to greater P2Y (12) blockade and consequently greater inhibition of platelet aggregation
Lau et al. [78]	2011	USA	Case–control	Postcoronary stent patients	Clopidogrel	St John's Wort	SJW may be a future therapeutic option to increase CYP metabolic activity and antiplatelet effect of clopidogrel in hyporesponders
Berger et al. [79]	2009	USA	Case–control	Patients with cardiovascular disease	Clopidogrel	Smoking	Clopidogrel therapy may be more effective in current smokers, but it may also confer a greater bleeding risk than in nonsmokers
Liu et al. [80]	2020	China	Meta‐analysis	Patients with acute coronary syndrome	Clopidogrel	Smoking	A significantly lower residual platelet reactivity in current smokers compared to that in nonsmokers
Ramatowski et al. [81]	2020	Poland	Case–control	Patients after PCI	Clopidogrel	Smoking	Smoking cessation in clopidogrel‐treated patients after PCI is associated with increased platelet reactivity and greater risk of HPR
Steinhubl et al. [83]	2008	USA	Case–control	Healthy volunteer	Clopidogrel	Cangrelor	The sustained platelet inhibition anticipated for clopidogrel treatment did not occur when cangrelor was initiated simultaneously
Lev et al. [84]	2007	USA	Case–control	Healthy volunteer	Clopidogrel	Caffeine	Acute caffeine administration after clopidogrel loading appears to be associated with enhanced platelet inhibition 2–4 h after clopidogrel intake
Angiolillo et al. [85]	2008	USA	Case–control	T2DM patients on dual antiplatelet therapy	Clopidogrel	Cilostazol	Adjunctive treatment with cilostazol in T2DM patients on standard dual antiplatelet therapy enhances inhibition of platelet P2Y(12) signaling
Farid et al. [87]	2009	USA	Case–control	Patients with ACS	Prasugrel	Rifampin	Formation of prasugrel's active metabolite is not affected by potent enzyme induction with rifampin
Small et al. [90]	2008	USA	Case–control	Healthy volunteer	Prasugrel	Lansoprazole	In subjects with high IPA after a clopidogrel dose alone that lansoprazole decreased IPA, whereas IPA was unaffected in these same subjects after a prasugrel dose
Wei et al. [91]	2022	China	Case–control	Patients after PCI	Ticagrelor	PPIs H2 receptor antagonist	The short‐term combination therapy with ticagrelor and PPIs or H2RA is safe and effective in patients with acute STEMI after PCI
Yan et al. [92]	2016	China	Retro	Patients after PCI	Clopidogrel Ticagrelor	PPIs	In patients with ACS following PCI, concomitant use of PPIs was not associated with increased risk of adverse outcomes in patients receiving either clopidogrel or ticagrelor
Nicolau et al. [93]	2015	Brazil	Case–control	Patients with ACS	Prasugrel Clopidogrel	PPIs	Use of PPIs did not result in a differential antiplatelet response between prasugrel versus clopidogrel but was associated with a lower incidence of MI with prasugrel
Hoedemaker et al. [94]	2019	Netherlands	Case–control	Patients with ACS	Prasugrel Clopidogrel	PPIs	PPI treatment at discharge was associated with a reduction in death, MI, or stroke at 30‐days postdischarge, mainly driven by a reduction in MI
Saad et al. [101]	2020	Germany	Case–control	Patients with MI	Ticagrelor	Morphine	Metoclopramide coadministration with morphine positively influences pharmacokinetics and pharmacodynamics of ticagrelor and may have rescue effects on the morphine/ticagrelor interaction
Buszko et al. [104]	2021	Poland	Case–control	Healthy volunteer	Ticagrelor	Morphine	Morphine delays the time of maximum drug concentration of ticagrelor and that the morphine effect occurs due to decreased gastrointestinal motility
Matsikas et al. [102]	2024	USA	Retro	People living with human immunodeficiency virus	Prasugrel Clopidogrel Ticagrelor	Antiretrovirals	High incidence of DDI between P2Y12inh and ART in PLWH, MACE and bleeding events at 1 year did not correlate with DDI
Kim et al. [105]	2021	Korea	Case–control	Rat	Ticagrelor	Dronedarone	A potential interaction between dronedarone and ticagrelor in humans is justified
Goli et al. [106]	2019	Ireland	Case–control	Patients after coronary angiography	Ticagrelor	Fentanyl	Fentanyl slows the absorption of oral ticagrelor, attenuating its effect on platelet inhibition
Ibrahim et al. [107]	2018	USA	Case–control	Patients required coronary stenting	Ticagrelor	Fentanyl	Fentanyl administration can impair ticagrelor absorption and delay platelet inhibition, resulting in mild excess of myocardial damage
Iglesias et al. [108]	2020	Switzerland	Case–control	Patients with MI	Ticagrelor	Fentanyl Morphine	Fentanyl did not improve platelet inhibition at 2 h after ticagrelor LD administration in comparison with morphine in patients with STEMI
Tavenier et al. [109]	2022	Netherlands	Case–control	Patients with MI	Ticagrelor	Fentanyl	The iv acetaminophen in comparison with iv fentanyl was not associated with significantly lower platelet reactivity in STEMI patients but resulted in significantly higher ticagrelor plasma levels
Uchiyama et al. [112]	2021	Japan	Case–control	Patients with AIS	DAPT (aspirin plus clopidogrel or cilostazol)	NA	DAPT using cilostazol was superior to SAPT with clopidogrel or aspirin for the prevention of recurrent stroke and vascular events without increasing bleeding risk among patients with AIS
Wantanabe et al. [113]	2022	Japan	Case–control	Patients with acute coronary syndrome	DAPT (aspirin plus clopidogrel)	NA	Clopidogrel monotherapy after 1–2 months of DAPT failed to attest noninferiority to standard 12 months of DAPT for the net clinical benefit with a numerical increase in cardiovascular events despite reduction in bleeding events
Genus et al. [114]	2022	France	Case–control	Patients with ACS	DAPT (aspirin plus clopidogrel)	NA	Compared with 4 months of DAPT, 12 months of DAPT does not reduce the risk of ACS recurrence, but increases the risk of bleeding
Li et al. [115]	2024	China	Case–control	Patients after PCI	DAPT (aspirin plus clopidogrel)	NA	An extended 9‐month clopidogrel monotherapy regimen was superior to continuing DAPT with clopidogrel in reducing clinically relevant bleeding without increasing ischemic events
Wu et al. [123]	2023	China	Case–control	Patients undergoing coronary drug‐eluting stent implantation	DAPT (aspirin or indobufen plus clopidogrel)	NA	Indobufen plus clopidogrel DAPT compared with aspirin plus clopidogrel DAPT significantly reduced the risk of 1‐year net clinical outcomes, which was driven mainly by a reduction in bleeding events without an increase in ischemic events
Wang et al. [124]	2022	China	Case–control	Patients with minor stroke or transient ischemic attack	DAPT (aspirin plus clopidogrel)		The risk of bleeding was greater in nonsmoking patients, and was associated with treatment with ticagrelor‐aspirin compared with clopidogrel‐aspirin

Abbreviations: ACS, acute coronary syndrome; AF, atrial fibrillation; AIS, acute ischemic stroke; ART, antiretrovirals; CCBs, calcium channel blockers; CHD, coronary heart disease; DAPT, dual antiplatelet therapy; DDI, drug–drug interaction; DOAC, direct oral anticoagulants; HPR, high platelet reactivity; IPA, inhibition of platelet aggregation; IS, ischemic stroke; MACEs, major adverse cardiovascular events; MI, myocardial infarction; NSAID, non‐steroidal anti‐inflammatory drugs; OTC, over‐the‐counter drug; PCI, percutaneous coronary intervention; PLWH, people living with human immunodeficiency virus; PPI, proton pump inhibitor; STEMI, ST‐elevation myocardial infarction; T2DM, type 2 diabetes mellitus; VTE, venous thromboembolism.

## 3 Aspirin

### 3.1 Antiplatelet Mechanisms of Aspirin

**FIGURE 2:**
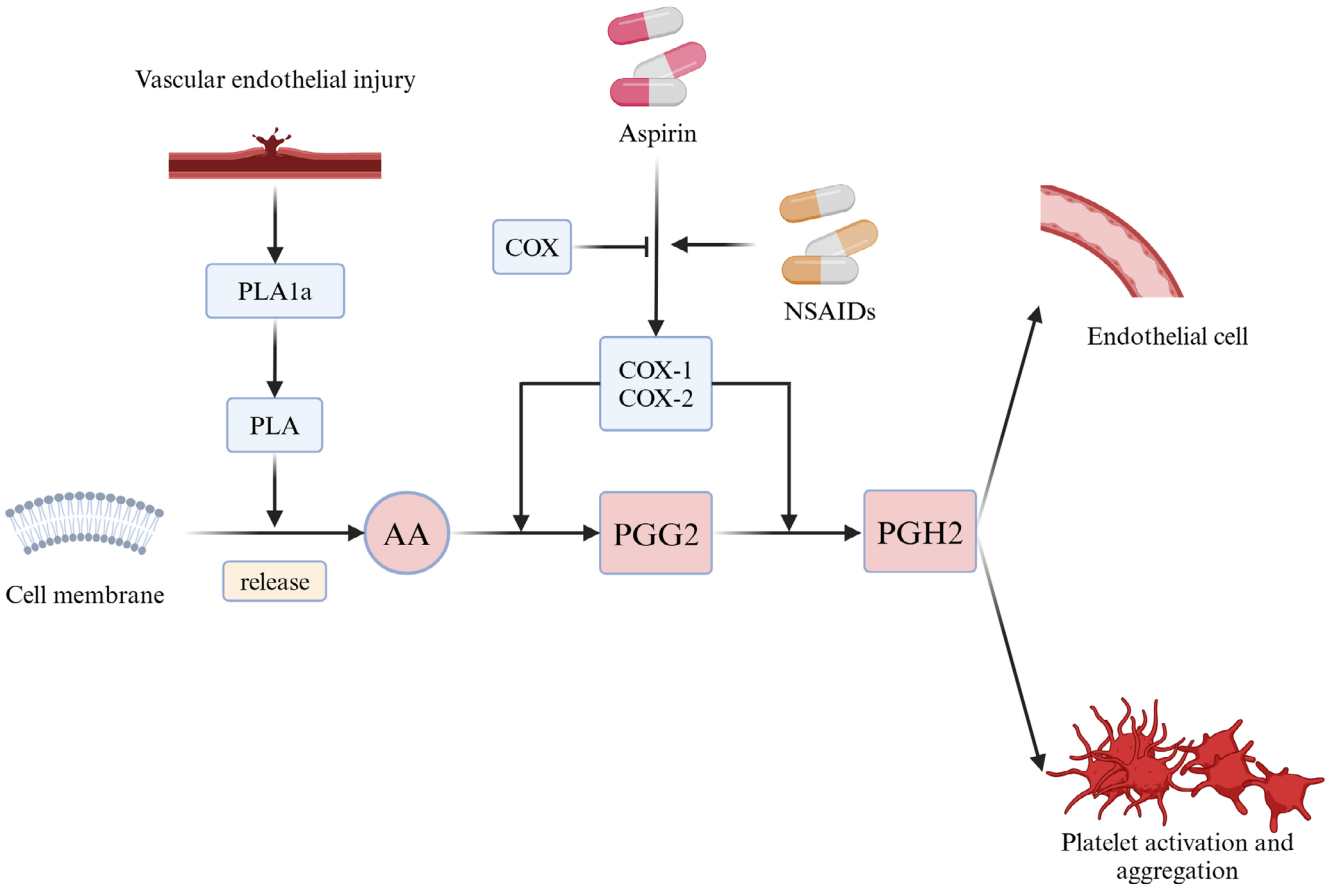
Mechanisms of aspirin‐drug interaction. AA, arachidonic acid; COX, cyclooxygenase; PGH2, prostaglandin H2; PGG2, prostaglandin G2; TxA2, thromboxane A2.

Aspirin (acetylsalicylic acid) is one of the most widely used treatments for ASVD and has been recognized for its antiplatelet properties since the 1950s [7, 8, 9]. During the acute phase of ASVD, arachidonic acid (AA) is released from vascular endothelial cells and metabolized by cyclooxygenase enzymes (COX‐1 and COX‐2) to form prostaglandins (PGG2 and PGH2). These intermediates are further converted into thromboxane A2 (TxA2) by thromboxane synthase in platelets and into other prostaglandins (PGD2, PGE2, and PGI2) by prostaglandin synthase in endothelial tissues (Figure 2) [10, 11].

Aspirin irreversibly inhibits COX‐1 activity, reducing serum TxA2 levels and thereby preventing platelet aggregation [12]. Additionally, aspirin has direct inhibitory effects on platelet aggregation through mechanisms independent of COX inhibition, further enhancing its antiplatelet action.

### 3.2 Platelet Function Tests

Significant variations in aspirin pharmacodynamics were first noted in the 1990s with the identification of aspirin resistance (AR) —a phenomenon characterized by high residual platelet reactivity despite aspirin therapy [13]. AR is typically detected using platelet function tests, which have since evolved to assess aspirin responsiveness accurately (Table 2). Research indicates that the prevalence of AR varies with laboratory methodologies, aspirin dosage, and administration frequency [14, 15]. For instance, patients receiving daily doses of ≥ 300 mg exhibit a lower incidence of AR compared to those on ≤ 100 mg. Furthermore, methods such as platelet function analyzer (PFA)‐100 and platelet function analyzer (RPFA) report higher AR prevalence compared to other laboratory techniques [16].

**TABLE 2:** Summary of commonly used platelet function tests.

Test name	Advantages	Disadvantages	Clinical utility
Bleeding time	The vivo test Rapid No white blood processing	Low reproducibility Invasive Poorly standardized Variable	Low clinical utility to predict bleeding
TxA2 Metabolites	The vivo test	From sources other than the platelets	Monitoring the effect of aspirin
Optical Aggregometry	Gold standard Flexible	Complicated Time‐consuming Requiring large blood volume Requiring enough expertise Limited by platelet count and blood lipid	Diagnosis of platelet dysfunction/disorders Monitoring the effect of antiplatelet therapy Potential for assessing hyperaggregability
Platelet Function Analyzer	Simple Rapid Require small blood volume Sensitive to severe platelet defects	Inflexible Requiring pipetting Limited by vWF Limited by HCT and platelet count Not sensitive to platelet secretion defects	Identification of various platelet disorders Exclusion of a variety of von Willebrand disease Monitoring the effect of P2Y12 inhibitors Monitoring the effect of aspirin
Impedance Aggregometry	Flexible Simple Sensitive to antiplatelet therapy	Expensive Limited by HCT and platelet count	Monitoring the effect of antiplatelet therapy Identification of various platelet disorders Identification of patients with a bleeding diathesis Identification of patients at risk of blood loss pre‐ or intraoperatively
Flow Cytometry Methods	Flexible Require small blood volume	Expensive Requiring specialized expertise	Assessment of platelet activation state Monitoring the thrombopoiesis Diagnosis of platelet glycoprotein defects Diagnosis of platelet dysfunction Monitoring the effect of P2Y12 inhibitors
VerifyNow	Simple Rapid High reproducibility	Inflexible Expensive Limited by HCT and platelet count	Monitoring the effect of antiplatelet therapy
Global thrombosis test	Global platelet method Require small blood volume	Lack of clinical studies Not widely available	
Plateletworks	Rapid Require minimal blood volume	Requiring adjunctive platelet count	
ROTEM Platelet Test	Rapid	Immediate Limited by HCT and platelet count Lack of clinical studies	Predicting the risk of excessive postoperative bleeding Predicting the need for blood products

Abbreviations: HCT, hematocrit; LTA, Light transmission platelet aggregation; ROTEM, rotational thromboelastometry; TxA2, thromboxane; vWF, von Willebrand factor.

### 3.3 Drug Interactions Between Aspirin and Anticoagulants

Aspirin is frequently coadministered with anticoagulants in patients with conditions such as atrial fibrillation, acute coronary syndrome, and cardiogenic stroke [17, 18, 19, 20].

When combined with warfarin, a widely used oral anticoagulant that inhibits vitamin K regeneration to reduce clotting factor synthesis [21, 22], aspirin increases the risk of bleeding without providing significant additional efficacy in reducing ASVD events [23, 24, 25, 26]. This interaction arises because aspirin displaces warfarin from plasma protein binding sites, thereby elevating free warfarin levels and amplifying its anticoagulant effect [23]. Additionally, studies have shown that this combination may prolong warfarin's time in the therapeutic range (TTR), further heightening bleeding risks [27].

Similarly, novel oral anticoagulants (NOACs), such as apixaban, dabigatran, edoxaban, and rivaroxaban, which inhibit thrombin or factor Xa, have been associated with increased bleeding and hospitalization rates when used concurrently with aspirin, without a corresponding reduction in ASVD incidence [28, 29]. The exact mechanisms underlying the interaction between aspirin and DOACs remain unclear, necessitating further investigation.

### 3.4 Drug Interactions Between Aspirin and Other NSAIDs

Nonsteroidal anti‐inflammatory drugs (NSAIDs) can reduce aspirin's antiplatelet efficacy by preventing its irreversible inhibition of platelet COX‐1 activity (Figure 2) [30].

Key factors influencing this interaction include the doses and timing of aspirin and NSAIDs (e.g., low‐dose ibuprofen or single‐dose naproxen) administration [31, 32, 33]. Higher aspirin doses and more frequent administration have been shown to mitigate this effect; for instance, a daily dose of 300 mg enhances platelet inhibition in patients with type 2 diabetes mellitus (T2DM) compared to a 100 mg dose [34]. Similarly, administering 100 mg aspirin twice daily achieves better platelet inhibition than a single 200 mg daily dose [35]. Among NSAIDs, celecoxib is considered safer when combined with aspirin, as it is associated with fewer gastrointestinal and renal adverse events compared to ibuprofen or naproxen. However, the addition of aspirin diminishes the safety advantage of celecoxib [36]. To maximize aspirin's antiplatelet efficacy in preventing ASVD, it is recommended to administer aspirin prior to NSAID use [37].

### 3.5 Drug Interactions Between Aspirin and Other Drugs

Aspirin interacts with various medications and natural compounds, potentially altering its pharmacokinetics and efficacy. Amoxicillin, for example, has been shown to slow the gut metabolism of aspirin's active metabolite, salicylic acid, thereby modifying its pharmacokinetic profile [38]. Natural medicines such as *Ginkgo biloba* extract (GBE) can influence platelet aggregation and increase bleeding risk when combined with aspirin [39].

Furthermore, selective serotonin reuptake inhibitors (SSRIs), widely used as antidepressants, have been found to elevate bleeding risk when coadministered with aspirin. This effect is likely mediated by the antiplatelet properties of SSRIs and their protective influence on the endothelium [40, 41, 42, 43].

## 4 Clopidogrel

### 4.1 Antiplatelet Mechanisms of Clopidogrel

**FIGURE 3:**
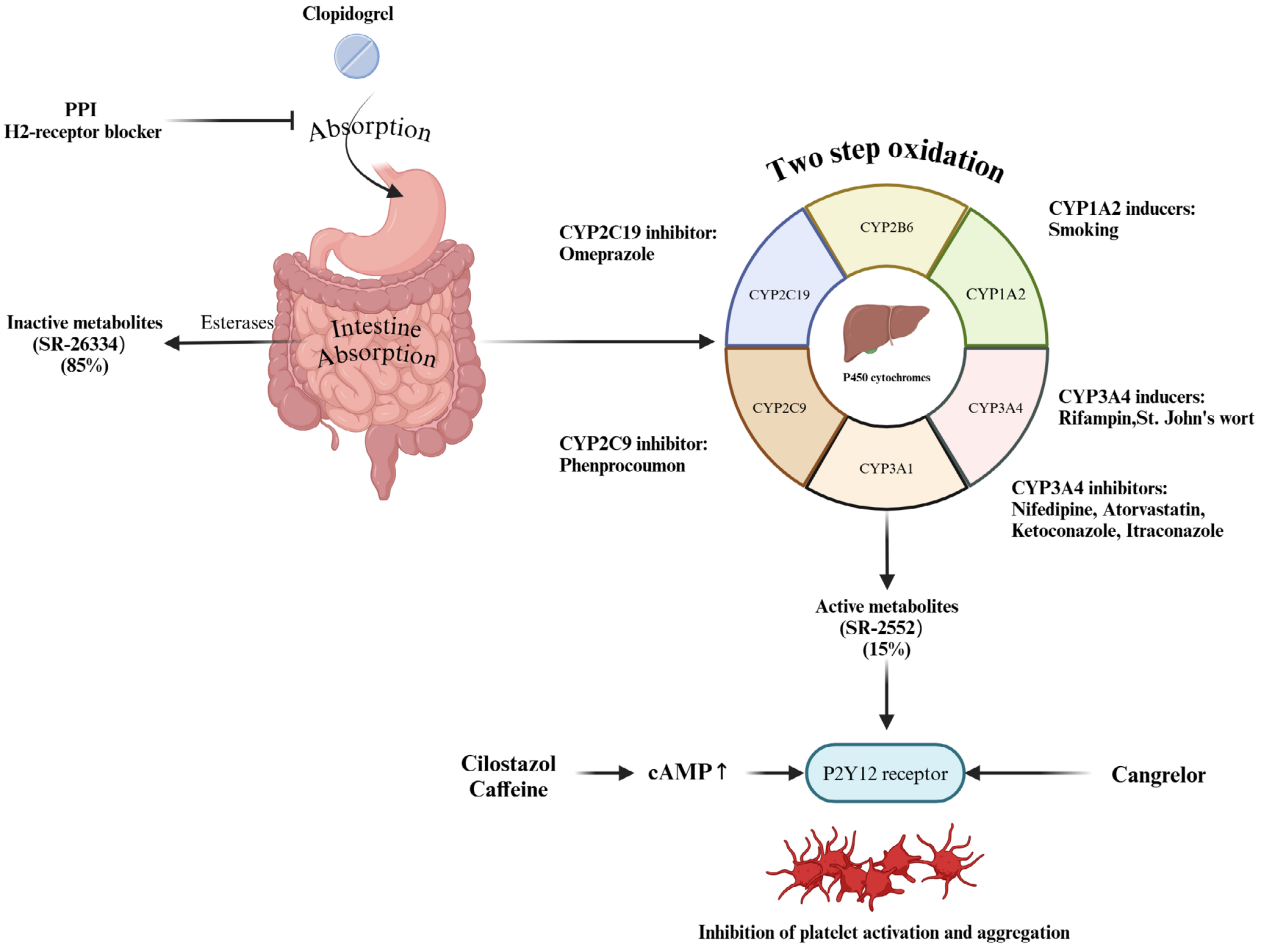
Mechanisms of clopidogrel‐drug interaction. PPI, Proton pump inhibitor.

Clopidogrel, a second‐generation thienopyridine, is the most commonly used ADP receptor antagonist for antiplatelet therapy [44]. The majority of clopidogrel is metabolized by hepatic carboxylesterase 1 into inactive metabolites (SR‐26334), which are excreted through the gut. Only a small fraction undergoes a two‐step bioactivation via hepatic cytochrome P450 (CYP) isoenzymes to produce its active metabolites (SR‐2552), which binds to the P2Y12 receptor, effectively inhibiting platelet activation and aggregation (Figure 3) [45].

### 4.2 Drug Interactions Between Clopidogrel and Anticoagulants

While clopidogrel and anticoagulants theoretically increase bleeding risk when coadministered, evidence suggests no significant drug interaction between clopidogrel and warfarin [46]. However, NOACs may alter clopidogrel's antiplatelet activity. P‐glycoprotein (P‐gp) plays a key role in the metabolism of NOACs, and modulators of P‐gp can affect NOAC plasma concentrations [47]. Ji et al. [48] demonstrated in mice that reduced P‐gp activity correlates with enhanced production of clopidogrel's active metabolite H4, potentially impacting its clinical efficacy. Additionally, phenprocoumon may reduce clopidogrel's antiplatelet effect due to shared metabolic pathways involving CYP2C19 and CYP3A4 [49].

### 4.3 Drug Interactions Between Clopidogrel and Calcium Channel Blockers

Calcium channel blockers (CCBs), commonly prescribed for hypertension and heart rate control, can influence clopidogrel's antiplatelet efficacy by inhibiting CYP3A4, thereby reducing the production of its active metabolites (Figure 3) [50, 51]. Studies suggest that the CCB‐clopidogrel interaction may also involve single nucleotide polymorphisms (SNPs) in CYP3A5, rather than CYP2C19 [52, 53]. The accumulation of CYP3A4 (IVS10+12G>A) A‐alleles may intensify these interactions [54]. Among CCBs, dihydropyridine agents like amlodipine have a variable impact, as they do not inhibit P‐glycoprotein [55]. Cilostazol has been shown to counteract this interaction and restore clopidogrel's activity [56]. However, a large observational study has not conclusively demonstrated that CCBs significantly reduce clopidogrel's efficacy, indicating the need for further clinical investigation [57].

### 4.4 Drug Interactions Between Clopidogrel and Proton Pump Inhibitors

Proton pump inhibitors (PPIs) irreversibly inhibit gastric acid secretion and are commonly coprescribed with clopidogrel to reduce gastrointestinal bleeding risks [58, 59]. However, emerging evidence indicates that combining PPIs with clopidogrel in patients with ASVD may increase the risk of major cardiovascular events and recurrence of ASVD [60, 61, 62, 63]. This adverse interaction is thought to arise from PPIs interfering with clopidogrel absorption (Figure 3) and metabolism, contributing to clopidogrel resistance (CR). PPIs such as omeprazole and esomeprazole, which have a high affinity for CYP2C19, can significantly inhibit the formation of clopidogrel's active metabolites compared to pantoprazole or rabeprazole (Figure 3) [64, 65, 66]. Omeprazole, in particular, has been shown to exert time‐dependent inhibitory effects on the generation of clopidogrel's active metabolites, thereby diminishing its antiplatelet efficacy [64].

This interaction is further modulated by CYP2C19 genotypes. Rapid metabolizers (RMs, *1/*1) appear to experience suppressed platelet aggregation when coadministered omeprazole or rabeprazole, while decreased metabolizers (DMs, *2/*3 carriers) are less affected [67]. Evidence suggests that alternative strategies, such as using H_2_‐receptor antagonists like famotidine, may mitigate these interactions and better preserve clopidogrel's efficacy [68].

### 4.5 Drug Interactions Between Clopidogrel and Statins

Statins, essential for reducing morbidity and mortality in cardiovascular diseases, are known to stabilize atherosclerotic plaques and modulate inflammatory pathways [69].

However, potential interactions between statins and clopidogrel arise because both are metabolized by CYP3A4, leading to competition within this enzymatic pathway (Figure 3). Despite theoretical concerns, most clinical studies have reported no significant reduction in clopidogrel's antiplatelet activity when coadministered with CYP3A4‐metabolized statins [70, 71].

Nevertheless, genetic predispositions such as the presence of CYP2C19*2 alleles may heighten the susceptibility to these interactions [72]. Lau et al. [73] demonstrated a dose‐dependent attenuation of clopidogrel's inhibition of platelet aggregation (IPA) by atorvastatin in patients undergoing coronary artery stent implantation. Similarly, in vitro studies suggest that simvastatin may inhibit CES1‐catalyzed absorption of clopidogrel and its active metabolite [74]. The clinical impact of these interactions appears dose‐dependent [73, 75].

### 4.6 Drug Interactions Between Clopidogrel and Other Drugs

The metabolism of clopidogrel is highly susceptible to interactions with various drugs that act on liver P450 enzymes. For instance, ketoconazole, a CYP3A4 inhibitor, reduces clopidogrel's antiplatelet activity by interfering with the production of its active metabolites (Figure 3) [76]. Conversely, rifampicin, a CYP3A4 inducer, significantly enhances clopidogrel's IPA by stimulating enzyme activity and enhancing P2Y12 receptor blockade [77]. St. John's wort, another CYP3A4 inducer, has been shown to boost the production of clopidogrel's active metabolite, although this may increase bleeding risks [78]. Smoking, a well‐known inducer of CYP1A2, enhances clopidogrel's platelet inhibition, resulting in shorter ischemic times but also an elevated risk of bleeding compared to nonsmokers (Figure 3) [79, 80, 81]. The non‐nucleoside reverse transcriptase inhibitor (NNRTI) etravirenz inhibits both CYP 2C19 and CYP 2C9, which in turn reduces the bioactivity of clopidogrel [82]. Certain drugs act directly on the P2Y12 receptor or indirectly influence clopidogrel's activity. For example, cangrelor, a direct P2Y12 receptor antagonist, synergistically enhances platelet inhibition when used alongside clopidogrel (Figure 3) [83]. Cilostazol and caffeine, which elevate intracellular cAMP levels, indirectly potentiate clopidogrel's inhibition of platelet activity by antagonizing P2Y12 receptor signaling (Figure 3) [84, 85]. These interactions highlight the complexity of managing clopidogrel therapy in patients on multiple medications and underscore the need for individualized treatment plans.

### 4.7 Drug Interactions of Prasugrel

Prasugrel, a third‐generation thienopyridine, undergoes rapid absorption in the intestinal lumen, where it is hydrolyzed by esterase CES2 into its thiolactone metabolite R‐95913 [86, 87]. Unlike clopidogrel, prasugrel's pharmacokinetics and pharmacodynamics are relatively unaffected by CYP3A4 inhibitors (e.g., ketoconazole or ritonavir) or inducers (e.g., rifampin) [76, 88, 89]. This makes prasugrel a viable alternative in scenarios where coadministration of CYP3A4‐modulating drugs is unavoidable. However, potential interactions between prasugrel and PPIs remain a topic of debate. The PRINCIPLE‐TIMI trial observed a reduction in prasugrel's active metabolite concentration with lansoprazole coadministration [90]. Contrarily, other observational studies and randomized controlled trials (RCTs) reported no significant impact of PPI coprescription on prasugrel's efficacy [91, 92, 93, 94]. These conflicting findings highlight the need for further research to clarify the clinical implications of these interactions, particularly in patients at high risk for gastrointestinal complications.

## 5 Ticagrelor

### 5.1 Antiplatelet Mechanisms of Ticagrelor

Ticagrelor, a reversible inhibitor of the P2Y12 ADP receptor, has demonstrated superior efficacy in reducing thrombotic events compared to clopidogrel [95]. The PLATO trial confirmed ticagrelor's significant reduction in vascular death, myocardial infarction, and stroke among patients with acute coronary syndrome, establishing it as a preferred option for managing ASVD [96].

Unlike clopidogrel, ticagrelor does not require metabolic activation by the liver, making its pharmacokinetics unaffected byCYP2C19 polymorphisms. This characteristic is particularly advantageous in populations with a high prevalence of CYP2C19 loss‐of‐function alleles, such as Han Chinese patients, for whom ticagrelor has been shown to be more effective than clopidogrel in stroke prevention [97].

### 5.2 Drug Interactions of Ticagrelor

**FIGURE 4:**
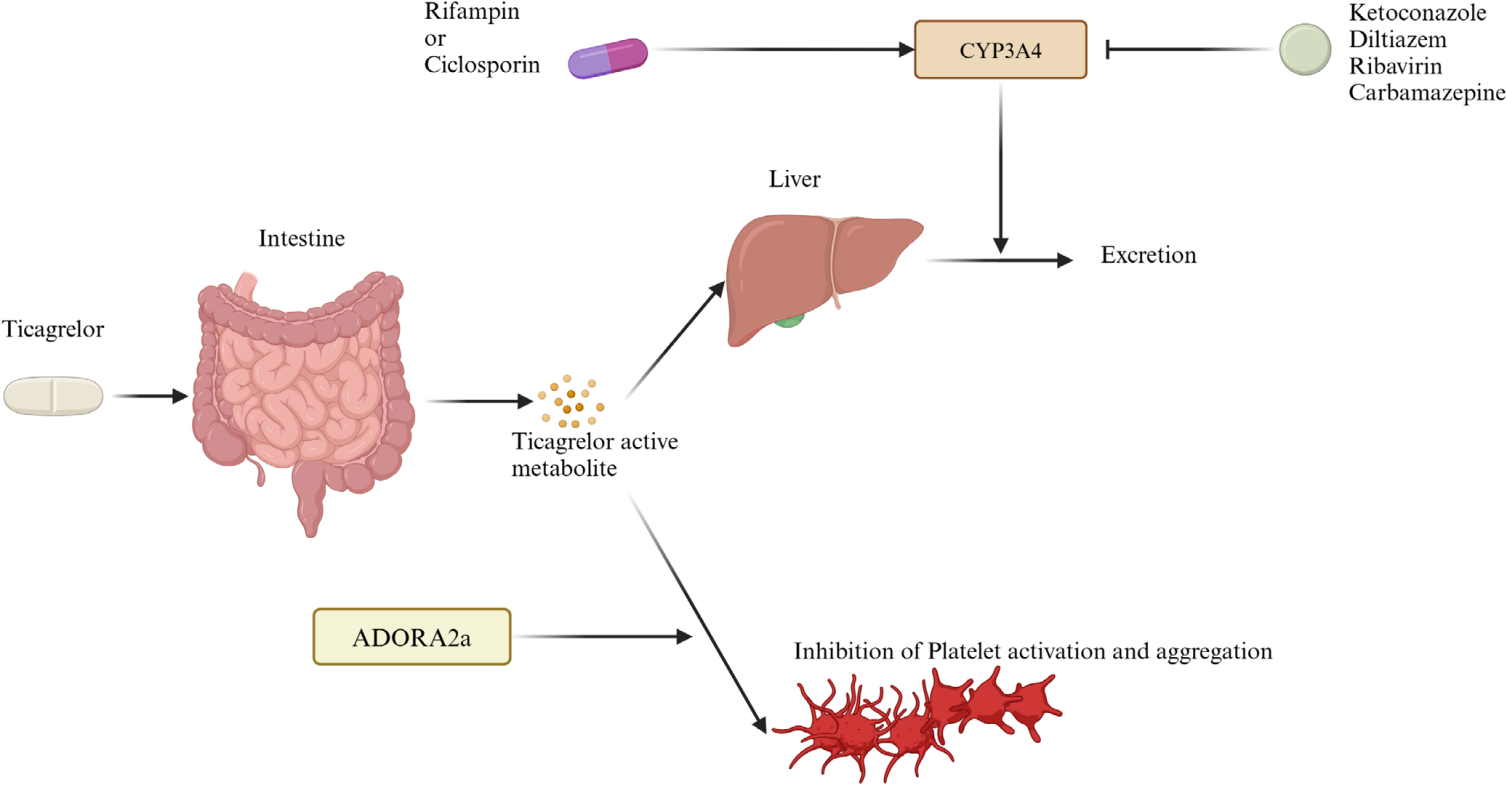
Mechanisms of ticagrelor‐drug interaction.

Ticagrelor is rapidly absorbed in the gastrointestinal tract and inactivated by CYP3A4 in the liver (Figure 4) [98]. Consequently, drugs that inhibit or induce CYP3A4 can significantly influence its pharmacokinetics. CYP3A4 inhibitors such as ketoconazole and diltiazem can slow the metabolism of ticagrelor, leading to elevated plasma concentrations and enhanced antiplatelet effects [99]. Conversely, CYP3A4 inducers like rifampin decrease ticagrelor's efficacy by reducing the levels of its active metabolites (Figure 4) [100]. Recent findings also highlight potential interactions with other drugs; for example, dronedarone significantly increased ticagrelor exposure in a rat model, although human studies are currently lacking (Figure 4) [101]. Similarly, ticagrelor coadministration with antiretroviral drugs like ribavirin has been associated with an increased risk of cardiovascular adverse events in HIV‐infected individuals [102], while carbamazepine has been shown to impair ticagrelor's antiplatelet activity by inducing CYP3A4 metabolism [103]. Beyond metabolic interactions, ticagrelor's absorption can be influenced by drugs affecting gastrointestinal motility. Morphine delays gastric emptying, prolonging the time to reach peak ticagrelor plasma levels and thus delaying its antiplatelet effect (Figure 4) [104, 105]. Interestingly, this mechanism does not affect prasugrel, highlighting the distinct pharmacodynamic profiles of these antiplatelet agents. These findings underscore the importance of careful monitoring and potential dose adjustments when ticagrelor is coadministered with drugs that can influence its metabolism or absorption, particularly in complex clinical settings.

## 6 DAPT

DAPT involves the concurrent use of two antiplatelet agents, most commonly aspirin and a P2Y12 inhibitor like clopidogrel or ticagrelor, to enhance antiplatelet efficacy and reduce the risk of thrombosis [106, 107]. However, this approach is associated with a higher bleeding risk. While DAPT is effective for preventing thrombotic events, studies have shown that long‐term DAPT does not significantly lower the recurrence of acute coronary syndrome compared to short‐term DAPT but is associated with a marked increase in bleeding events [108, 109, 110]. The bleeding risk varies depending on the combination of antiplatelet drugs used in DAPT. Cilostazol, a phosphodiesterase inhibitor with antiplatelet, vasodilatory, anti‐inflammatory, and anti‐atherosclerotic properties, has demonstrated a favorable safety profile [19, 111, 112]. Studies indicate that cilostazol does not significantly increase bleeding risk when used in combination with clopidogrel or aspirin, making it a safer alternative in specific patient populations [112].

Another option, indobufen, inhibits platelet aggregation by reversibly blocking platelet cyclooxygenase and suppressing thromboxane A2 biosynthesis [113, 114, 115, 116, 117]. Evidence suggests that combining clopidogrel with indobufen results in fewer bleeding events compared to the clopidogrel and aspirin combination [118]. In contrast, the combination of ticagrelor and aspirin, while effective in reducing thrombotic risks, carries a higher bleeding risk compared to clopidogrel‐based combinations [119]. These findings underscore the importance of individualizing DAPT regimens to balance the antithrombotic benefits and bleeding risks, particularly in high‐risk patients.

## 7 Discussion

In clinical practice, DDIs between antiplatelet drugs and other medications are well‐documented. Although strategies to address drug resistance have been proposed, further robust research is needed to establish optimal management approaches for resistance and to ensure the safety and efficacy of antiplatelet therapy.

### 7.1 Classification of Clinical Settings for DDIs

Antiplatelet drug interactions vary significantly depending on the clinical setting, requiring tailored strategies for each scenario.

#### 7.1.1 Outpatient Long‐Term Management

In outpatient settings, patients with ASVD often receive a combination of antiplatelet drugs, statins, antihypertensive agents, and other cardiovascular medications. These combinations are prone to DDIs that may alter drug metabolism and reduce platelet inhibition efficacy, particularly with statins or CCBs. Regular monitoring of liver function, platelet count, and clinical signs of bleeding is crucial. A comprehensive evaluation of a patient's comorbidities should guide the selection of drug combinations to mitigate risks.

#### 7.1.2 Inpatient Emergency Care

Patients with acute myocardial infarction or other thrombotic emergencies are frequently treated with DAPT combined with thrombolytics or anticoagulants. In this setting, the primary challenge lies in balancing the prevention of thrombosis against the risk of bleeding. Potential risks include increased bleeding complications and altered responses to anticoagulants or thrombolytics. Effective management strategies involve regular monitoring of coagulation parameters, appropriate dose adjustments, and avoiding high‐risk drug combinations, such as ticagrelor with strong CYP3A4 inhibitors like ketoconazole.

#### 7.1.3 Neuro‐Intensive Care Unit Setting

In the neuro‐intensive care unit (ICU), patients with ischemic stroke often receive antiplatelet drugs in conjunction with other neuroprotective agents, anticoagulants, or analgesics. Interactions in this setting are particularly critical due to the elevated risk of intracranial hemorrhage.

Management requires frequent imaging to detect bleeding or ischemic progression, meticulous drug monitoring, and individualized dosing adjusted for renal and hepatic function. Avoiding combinations that elevate intracranial pressure or bleeding risk is vital.

### 7.2 Personalized Medication Related to Drug Interactions

A nuanced approach to personalized medication is essential in mitigating DDIs that may cause resistance to antiplatelet therapy. For example, modifications to aspirin dosing and frequency can address NSAID‐related AR [35], while administering a loading dose rather than a maintenance dose of clopidogrel has shown efficacy in counteracting atorvastatin‐induced CR [120].

Comprehensive assessment of patients' medication histories is critical to identifying potential DDIs and optimizing therapy.

Key recommendations include: (1) Monitor efficacy: Frequent evaluation of antiplatelet therapy's effectiveness is vital. For instance, AA‐induced platelet aggregation should be monitored in patients on aspirin or indobufen, while ADP‐induced aggregation should be checked in those receiving clopidogrel or ticagrelor. (2) Tailoring medication: Treatment should align with individual patient characteristics, such as age, comorbidities, and renal or hepatic function, to balance therapeutic benefits and risks. (3) Patient education: Empowering patients through education on medication adherence and potential DDIs, including the risks of over‐the‐counter drugs and herbal remedies, enhances safety and compliance. (4) Genetic testing: Testing for genetic polymorphisms, particularly those affecting CYP2C19, is increasingly utilized in clinical settings to guide antiplatelet therapy. For instance, CYP2C19*2 and *3* alleles are associated with reduced efficacy of clopidogrel, especially in Asian populations, where the prevalence of these polymorphisms is higher [121, 122]. Carriers of CYP2C1917, on the other hand, may benefit from enhanced clopidogrel activity [123, 124]. CR may also be influenced by alleles such as A, GA, AA, and GG+GA within the CYP2C19*2 polymorphism [125]. A recent retrospective study highlighted a potential correlation between AR and CR [126]. Furthermore, CYP2C19 polymorphism plays a predictive role in the interaction effects of atorvastatin and PPIs on clopidogrel's antiplatelet activity [64, 72].

The intricate interplay between genetic variants and drug interactions underscores the importance of personalized approaches to antiplatelet therapy. Table 3 outlines other polymorphisms associated with antiplatelet drug resistance, yet further research is essential to comprehensively understand the broader genetic determinants influencing efficacy and resistance in antiplatelet therapy.

**TABLE 3:** Polymorphisms of genes related to antiplatelet drug‐resistance.

Gene	Encoding protein	Related drugs	Polymorphism	Function
CYP2C19 gene	CYP2C19 enzyme	Clopidogrel	*1/*1(RM)	Key enzyme in both first step and second steps of clopidogrel metabolism
			*1/*2, *1/*3(DM)	
			*2/*2, *3/*3, *2/*3(PM)	
			*17/*17(UM)	
ABCB1 gene	ABCB1 protein	Aspirin	G2677T(rs2032582): CT/TT	Involved in the absorption of aspirin and clopidogrel in the intestine
		Clopidogrel	C3435T(rs1045642): CT/TT	
PON‐1 gene	Paraoxonase 1	Clopidogrel	DNA methylation of CPG4 in the PON1 promoter	Esterase involved in the first step of clopidogrel metabolism
P2Y12 gene	P2Y12 receptor	Aspirin	C34T	Platelet activation and aggregation on the surface of platelets
		Clopidogrel	G52T	
			iT744C	
			rs6809699	
CES‐1 gene	Carboxylesterase 1	Clopidogrel	A (− 816) C	Part of phase II metabolism
KDR gene	Kinase Insert Domain Receptor	Clopidogrel	rs1870377	NA
			rs12115090	
			rs2305948	
ABCC3 gene	ABCC3 protein	Clopidogrel	NA	Facilitates transport of conjugated drug metabolites across cell membranes
PEAR1 gene	Platelet Endothelial Aggregation Receptor‐1	Aspirin Clopidogrel	NA	A platelet transmembrane protein
Insulin secretion related gene	NA	Clopidogrel	RAPGEF4 (rs17746510)	NA
			GCG (rs564): carry A or G	
FMO3 gene	Flavin‐containing monooxygenases	Clopidogrel	FMO3 (rs1736557): AG allele	Monooxygenase involved in the first step of clopidogrel metabolism
CRISPLD1	Secreted protein rich in cysteine	Clopidogrel	CRISPLD1 (rs12115090): A>C	NA
ABCC2	Transport protein	Clopidogrel	ABCC2 rs717620: TT allele	Post‐transcriptional regulation and interaction with CYP2C19
COX gene	COX‐1 COX‐2	Aspirin	COX‐1 rs1330344: G allele COX‐2rs20417: 765G‐C	Formation of prostanoids from arachidonic acid
PLA1a gene	Phospholipase A1	Aspirin	PLA1/A2 allele	Hydrolyzes phospholipids into fatty acid
P2Y1 gene	P2Y purinoceptor 1	Aspirin	P2Y1 893CC genotype	An important role in platelet aggregation
ITGA2 gene	Integrin alpha‐2	Aspirin	ITGA2 rs1126643: 807C‐T	Platelet‐surface‐mediated signaling
		Ticagrelor	ITGA2B rs5911: T>G	
ALOX5AP gene	5‐lipoxygenase‐activating protein	Aspirin	ALOX5AP1 SG13S114T/A: AA allele	An important part in leukotriene synthesis
TBXA2R gene	Thromboxane receptor	Aspirin	CC genotype	Promotes platelet adhesion, aggregation, degranulation, and platelet‐induced blood clotting‐responses
CYP4F2 gene	Leukotriene‐B (4) omega‐hydroxylase 1	Aspirin Ticagrelor	CYP4F2 rs3093135: T allele CYP4F2*1*3	Involved in the metabolism of fatty acids
ADORA2a gene	The adenosine A_2A_ receptor	Ticagrelor	ADORA2a rs5751876: *3	Adenosine levels affect the antiplatelet activity of ticagrelor

Abbreviations: DM, decreased metabolizer; PM, poor metabolizer; RM, rapid metabolizer; UM, ultrarapid metabolizer; NA, not available.

### 7.3 Large Clinical Trials

Large clinical trials are crucial for advancing our understanding of DDIs and their impact on antiplatelet therapy. However, several challenges remain. First, observational studies provide valuable insights into potential correlations but often fail to establish causality. RCTs, although more rigorous, may still be affected by unmeasured confounders and are frequently constrained by high costs and logistical barriers. Additionally, outcomes such as noncardiogenic deaths or vascular interventions, which are unrelated to platelet function, may confound endpoint analyses in these trials. Another significant limitation is individual variability in drug response. Factors such as genetic polymorphisms, age, sex, and medication adherence contribute to highly variable outcomes, making it challenging to develop universal recommendations. Addressing these gaps requires a combination of innovative trial designs, such as adaptive trials, and the integration of real‐world evidence to complement traditional RCT data.

### 7.4 Development of Novel Antiplatelet Drugs

The development of new antiplatelet agents offers promising avenues for improving outcomes in ASVD. Tirofiban, a glycoprotein IIb/IIIa inhibitor, has demonstrated efficacy in preventing platelet aggregation during acute phases of ASVD [127]. Additionally, cilostazol, a phosphodiesterase inhibitor with anti‐atherosclerotic and vasodilatory properties, has been shown to reduce atherosclerosis progression without increasing bleeding risk, as highlighted in a recent meta‐analysis [128]. While these agents provide novel mechanisms of action, their interactions with existing medications have not been thoroughly studied. For instance, potential DDIs involving newer agents and standard antiplatelet drugs remain unexplored, underscoring the need for further preclinical and clinical studies. Confirming the safety, efficacy, and compatibility of these drugs in combination regimens will be essential for their broader adoption in clinical practice.

## 8 Limitations

This review has several limitations that should be considered when interpreting the findings. First, although we have focused on commonly used antiplatelet agents, such as aspirin, clopidogrel, prasugrel, and ticagrelor, novel antiplatelet therapies and combination regimens are rapidly emerging, but data on their clinical efficacy and drug interactions remain limited. The exclusion of these agents from this review may lead to an incomplete understanding of the broader landscape of antiplatelet therapy. Second, while we have attempted to summarize the clinical implications of DDIs in various settings, many of the referenced studies are limited by sample size, retrospective designs, or heterogeneous populations, which may impact the generalizability of their conclusions. Third, genetic testing and personalized medicine, though emphasized as crucial tools in optimizing therapy, remain underutilized in clinical practice, particularly in resource‐limited settings. The lack of widespread genetic testing limits the applicability of recommendations derived from studies in specific populations. Additionally, the effects of polymorphisms beyond CYP2C19 on drug interactions and resistance are not fully understood, necessitating further research to broaden our genetic insights. Fourth, this review has focused primarily on the context of ASVD and related conditions. Patients with other comorbidities, such as cancer or chronic inflammatory diseases, may experience different patterns of DDIs, which were not explored here. Lastly, the majority of the studies included were observational or based on real‐world evidence, leaving room for confounding factors that RCTs might better address. Future research should aim to conduct large‐scale, high‐quality trials to clarify the long‐term impact of DDIs and to assess emerging antiplatelet agents and strategies.

## 9 Conclusion

Antiplatelet therapy is a cornerstone in the management of ASVD, yet the clinical utility of these agents is frequently compromised by DDIs that alter their efficacy or safety. This review highlights the pharmacokinetic and pharmacodynamic mechanisms underlying these interactions and underscores their implications across outpatient, emergency, and intensive care settings. Personalized therapeutic strategies, informed by genetic testing, rigorous monitoring, and tailored drug selection, are essential for optimizing outcomes while minimizing adverse events.

Future research should prioritize large‐scale clinical trials to explore the interactions of newer antiplatelet agents and assess the long‐term efficacy of personalized approaches. By addressing these challenges, clinicians can better navigate the complexities of antiplatelet therapy, ultimately improving outcomes for patients with ASVD and related conditions.

## Author Contributions

X.H. wrote the first draft of the manuscript; X.H., X.Z., J.S., and M.W. performed the material preparation, data collection, and statistical analysis; D.Z., R.M., and X.J. contributed to imaging assessments; D.Z., R.M., and Y.D. wrote sections of the manuscript and contributed to manuscript revision.

## Conflicts of Interest

The authors declare no conflicts of interest.

## Data Availability

Data sharing is not applicable to this article as no new data were created or analyzed in this study.
